# The Food and Nutrition Security for Manitoba Youth (FANS) study: rationale, methods, dietary intakes and body mass index

**DOI:** 10.1186/s40795-022-00611-x

**Published:** 2022-10-20

**Authors:** Joyce Slater, Bhanu Pilli, Aynslie Hinds, Alan Katz, Marcelo L. Urquia, Julianne Sanguins, Chris Green, Jaime Cidro, Dan Chateau, Nathan Nickel

**Affiliations:** 1 Department of Food and Human Nutritional Sciences, University of Manitoba, Winnipeg, MB R3T 2N2 Canada; 2 Department of Community Health Sciences, Max Rady College of Medicine, University of Manitoba, Winnipeg, MB R3E 0W3 Canada; 3 Department of Family Medicine, Max Rady College of Medicine, University of Manitoba, Winnipeg, MB R3E 0W2 Canada; 4 Manitoba Metis Federation, Winnipeg, MB R3B 0J7 Canada; 5 Winnipeg Regional Health Authority, Winnipeg, MB R3B 1E2 Canada; 6 Department of Anthropology, University of Winnipeg, Winnipeg, MB R3B 2E9 Canada; 7 Research School of Population Health, Australian National University, Canberra, ACT 0200 Australia

**Keywords:** Nutrition, Eating, Diet, Adolescent, Manitoba, Canada

## Abstract

**Background:**

Good nutrition and access to healthy foods are essential for child growth and development. However, there are concerns that Canadian children do not have a healthy diet, which may be related to dietary choices as well as lack of access to healthy foods. The FANS (Food and Nutrition Security for Children and Youth) study examined the nutrition and food security status of youth in the province of Manitoba, Canada. This paper describes methods, dietary intakes, and body mass index for the FANS study.

**Methods:**

This cross-sectional study included 1587 Manitoba grade nine students who completed a self-administered web-based survey. Data was collected on demographic characteristics, dietary intake (24-h recall), food behaviors, food security, and self-report health indicators. Dietary data was compared to national dietary guidelines (Dietary Reference Intakes and Canada’s Food Guide). Mean and median nutrient and food group intakes were calculated with corresponding measures of variability. Chi-square tests compared percentage of respondents not meeting key nutrients and food groups. Significant differences in percentage of total servings for each food group were determined by a Kruskal–Wallis test, and differences between different caloric groups were assessed using Dunn’s test for post-hoc comparisons.

**Results:**

Half of study respondents were female (50.5%). Median energy intake was higher in males (2281 kcal) compared with females (1662 kcal), with macronutrient distribution of 52%, 16%, and 32% for carbohydrates, protein, and fats respectively. Most participants consumed inadequate fibre (94%), vitamin D (90%), and calcium (73%), while median sodium intakes exceeded recommendations for males but not females. A majority of participants did not meet Health Canada’s recommendations for food group servings: Vegetables and Fruit (93%), Milk and Alternatives (74%), Meat and Alternatives (57%) and Grain Products (43%). Other Foods, including sugar sweetened beverages and juice, were consumed by most participants. Higher energy consumers had a greater proportion of food servings coming from Other Foods. 72.1% of students were classified as having a healthy weight and 25% were classified as overweight or obese.

**Conclusion:**

Poor dietary intakes and body mass index values indicate an urgent need for policy and program strategies to support healthy eating habits and food awareness in Manitoba youth.

## Background

Diet is the largest contributor to mortality at a population level, ahead of other risk factors such as inactivity and smoking [1]. Food insecurity and nutrition insecurity are both strongly linked with poor diet (nutrition insufficiency) [2, 3]. Food security is the ability to access food without economic constraints [4]. Nutrition security includes addressing hunger, but also requires that foods contain sufficient nutrients, and people consume those foods rather than highly processed, low-nutrition foods [5]. Nutrition insecurity impacts populations who are food insecure and food secure; therefore, with continued growth in nutrition-sensitive conditions, both dimensions of food and nutrition security require addressing [6].

Poor diet is linked with a variety of poor health outcomes, including noncommunicable diseases (NCDs): type 2 diabetes, cardiovascular disease, cancer and respiratory disease account for over 80% of premature NCD deaths globally [7]. Type 2 diabetes is the most prominent NCD linked to diet [8, 9], and it is increasingly affecting young adults. One in ten Canadians over the age of one have been diagnosed with diabetes, and between 2000–2016 young adults aged 20–34 experienced a 41% increase [10]. Food insecure Canadians have more than double the risk of developing type 2 diabetes compared to food secure Canadians [11] and food insecure youth are at higher risk of developing mental health problems [12]. In 2021, 15.9% of Canadian households experienced food insecurity, including more than 1.3 million children [13].

The dietary risk factors for NCDs are well-established: calorie-dense diets high in sugar, salt, and fat primarily from ultra-processed foods, and low in fruits, vegetables, lean proteins and whole grains lead to increased body weight, high blood pressure, elevated blood lipids and glucose [14, 15]. There is increasing evidence that obesogenic diets are facilitated by food and social environments that support access to affordable highly processed food [16]; yet paradoxically promote the ‘thin ideal’ [17]. In Canada highly processed and fast food are consumed regularly, and are associated with higher body mass index [18–20].

Of particular concern are the dietary intakes of Canadian children and youth, many of whom appear to be on track for developing obesity and NCDs at earlier ages. Measured heights and weights from the 2019 Canadian Health Measures Survey indicate that 10.1% of children ages 5 to 17 are obese [21] and by 2030 it is predicted that 15% of children age 10–19 will be obese [22], with poor diet a significant risk factor. There are few studies of dietary intake in Canadian children and youth; however, evidence suggests that overall diet quality is poor [23, 24]. Further, 43% of adolescent Canadian girls report body weight dissatisfaction [25].

Canada has recently committed to a national food policy [26], including addressing food insecurity and a new food guide [27], setting the stage for population dietary health to become a key goal across diverse policy agendas. Adolescence is a critically important developmental stage for building awareness and behaviours that can promote nutritional health into adulthood. It is therefore critical that an empirical knowledge base of youth dietary habits be developed to inform programmatic and policy strategies aimed at improving diet quality. The purpose of this research, ‘Food and Nutrition Security for Manitoba Youth (FANS)’ is to create a knowledge base to support the development of adolescent nutrition policy and programming in the province of Manitoba, Canada. The FANS study collected information on the dietary intakes, food behaviors, food security status, self-reported health indicators and body mass index of a cohort of grade 9 students, as well as information on socio-demographic factors relevant to contextualizing observed dietary trends. This initial paper describes the methods for the FANS study, as well as dietary intakes and quality, and body mass index for all participants*.* Future papers will analyze remaining variables.

## Methods

### Setting

The study took place in the province of Manitoba, Canada which has a population of 1,278,365 [28]. The capital city of Winnipeg is the largest urban centre with 61% of the provincial population. The percentage of recent immigrants, or newcomers (5.1%), who arrived between 2011 and 2016 is almost 50% higher than the rest of Canada [29]. Manitoba has the highest provincial child poverty rate in Canada: 28.3% compared to 18.2% nationally [30].

### Design and sampling

**Fig. 1:**
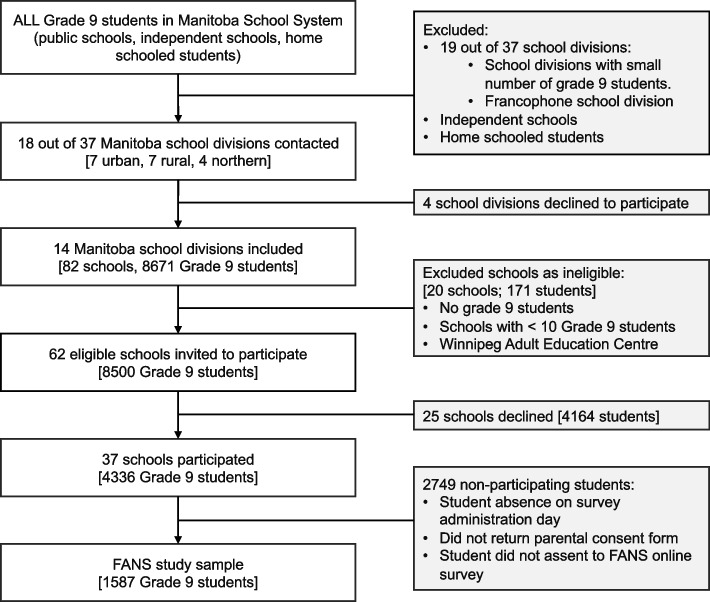
FANS sampling strategy

Using a cross-sectional design, the study collected dietary intake, food behaviour and food security data using a web-based survey, from grade 9 students using a stratified two-stage sampling method as follows. First, the largest 18 of 37 provincial public school divisions were approached by email and follow-up phone calls to participate. Nineteen school divisions were excluded due to small numbers of grade 9 students and prohibitive data collection (travel) costs. Fourteen school divisions chose to participate, and all classes with ≥ 10 grade 9 students were approached. Participating class teachers distributed parent/guardian consent forms to all students. All students for whom consent was received had the opportunity to assent at the beginning of the online survey. The final study sample was 1587 participants, which represented 37% of eligible students in the 37 participating schools. Sampling details are outlined in Fig. 1. Data were collected between October 2018 and May 2019.

### Survey measures

#### Demographic

Demographic characteristic questions asked for participant age, sex/gender (male/female/other), self-reported Indigenous ancestry (First Nations, Métis, Inuit, don’t know) and age of arrival if not born in Canada. Indigeneity questions were developed with Indigenous academic and community partners. “Newcomer” was defined as being born outside Canada and moving to Canada within the past 7 years; this construct was developed with academic and settlement organization partners.

Participating school divisions, and respective schools, were classified into rural, urban and northern regions of the province, reflecting existing indicators of these classifications [31, 32].

#### Dietary intake/food behaviors/health indicators/food security

Dietary intakes, food behaviours and health indicators were collected using the WEB-Q (Waterloo Eating Behaviour Questionnaire) [33]. WEB-Q is a valid, reliable tool for measuring the food and nutrient intake of adolescents using a 24-h dietary recall and food frequency questionnaire. The survey was administered on-site at schools and overseen by a research coordinator. Dietary intake was assessed through a 24-h recall module where students chose from a list of approximately 800 food items. Segmented into meals and snacks, students chose foods and beverages, and portion sizes, from pictures and associated text on the screen. A similar food was chosen if the original was not on the list. In addition to the recall, participants were asked about consumption frequency of sugar sweetened, caffeinated, and high protein beverages. Food behaviour assessment included questions about frequency of meal consumption, meals consumed with family members and food purchasing habits. Health indicators included questions about height and weight, attempts to lose or gain weight, and self-reported health and life satisfaction. Body mass index (BMI) was calculated using self-reported height and weight, and classified using World Health Organization z-scores [34]. Food security was assessed using the Child Food Security Survey Module validated for youth over 12 years of age [35]. The module consists of nine questions focused on access to food, concerns about food availability, modified eating behaviours, and hunger levels within the past twelve months [35].

### Administrative data linkage

A sub-set of students (*n* = 960) provided consent for FANS researchers to access de-identified provincial administrative health and socio-economic data using the student’s unique health number, through a Repository at the Manitoba Centre for Health Policy, University of Manitoba [36]. Dietary and survey data will be linked to income (neighbourhood level), mental health, housing and educational outcomes at the individual participant level and modelled for associations at the study population level in future papers [37].

### Data analysis

Nutrient intakes were assessed using ESHA software (Salem, OR, 2002) [38] and the Canadian Nutrient File database [39]. Intakes were compared with Dietary Reference Intakes (DRI) [40]. Inadequacy estimates were calculated for nutrients using the Estimated Average Requirement approach (EAR) [41–45]. Adequate Intake (AI) was used for dietary fibre where EAR was not available [46]. Excess sodium intakes were assessed based on tolerable upper intake levels (UL) [44]. Macronutrients were compared with the Acceptable Macronutrient Distribution Ranges (AMDR) [46]. Food groups and servings were compared with Eating Well with Canada’s Food Guide (2007) (EWCFG) [47], which were based on portion sizes from Canadian Nutrient File definitions and databases [48].

Overall, 1571 students completed the diet recall. Records were manually checked for quality during the data collection period. Final line-level data were imported into SPSS software and verified for missing and biologically implausible values. Students’ dietary intake data were excluded from analyses if they consumed less than 200 kcal/day or exceeded 6000 kcal/day (*n* = 35) [49] which resulted in a final analytical sample of 1536. Records where a meal-specific food item was selected four times or more (due to slow internet connection in a few data collection sites) were visually inspected and deemed to represent an implausible selection of the item, as the ≥ 4 meal-specific food selections were inconsistent with other food selections for these records. This occurred in 28 instances and the intake was adjusted to 2 servings (the median number of servings chosen by the sample for the meal-specific food items in question).

Study data were analyzed using SAS version 9.4 (variable derivation) and SPSS version 27 (tables and statistical outputs) statistical software packages. Mean and median nutrient and food group intakes were calculated with corresponding measures of variability. Chi-square tests were performed to compare percentage of males and females not meeting key nutrient recommendations, and food group recommendations, and Cramer’s V was used as a measure of effect size. Data was checked for normality using the Shapiro–Wilk test. Significant differences in percentage total servings for each food group were determined by a Kruskal–Wallis non-parametric test and differences between the caloric intake groups were assessed using Dunn’s test for post-hoc comparisons. Statistical significance was accepted at *p* ≤ 0.05. All analyses were undertaken using unweighted sample data.

## Results

### Demographic characteristics

Half of study respondents (50.5%) were female and 44.7% were male, with 4.8% of study respondents self-identifying as “other” or not disclosing their sex (Table 1). These respondents were excluded from sex-dependent analyses. Participants ranged in age from 13–16 years with the majority (95.9%) in the 14- and 15-year-old categories. Over three-quarters (77.5%) were from urban areas of Manitoba.

**Table 1:** Characteristics of study participants (*n* = 1587)

Demographics	Frequency, *n*	Percentage, %
Gender^a^		
Female	802	50.5
Male	709	44.7
Other	10	0.6
Not reported	66	4.2
Age^a^		
13 years	54	3.4
14 years	1296	81.7
15 years	225	14.2
16 years	8	0.5
Not reported	4	0.3
Region		
Northern	134	8.4
Rural	223	14.1
Urban	1230	77.5

^a^Self-report

### Nutrient intakes

Median energy intake for males was 2281 kcal and 1662 kcal for females (Table 2). One quarter of females consumed less than 1199 kcal while one quarter of males consumed less than 1736 kcal.

**Table 2:** Median nutrient intakes and interquartile ranges

Energy or Nutrient	Current Intake Guidelines	Total (*n* = 1536 recalls)			Females (*n* = 782 recalls)			Males (*n* = 682 recalls)		
		25^th^	Median	75^th^	25^th^	Median	75^th^	25^th^	Median	75^th^
Energy (kcals)		1385.6	1962.0	2608.9	1199.3	1661.7	2248.3	1735.6	2281.0	2916.2
Nutrients										
Carbohydrates (g)	100^c^	174.9	249.9	328.8	152.5	216.0	293.7	211.9	284.5	374.4
Sugar^a^ (g)		44.2	77.0	114.9	38.8	65.8	102.6	52.6	90.1	134.3
Fibre (g)	26^de^, 38^df^	9.2	14.3	20.6	8.4	12.7	18.5	10.5	16.1	23.3
Fat (g)		43.7	68.7	100.5	37.5	58.0	82.9	56.4	84.7	117.5
Saturated fat (g)		14.6	24.2	36.6	12.7	20.5	29.7	19.3	29.6	43.0
Monounsaturated fat (g)		13.9	22.6	34.0	12.5	19.4	28.4	18.4	27.6	41.8
Polyunsaturated fat (g)		7.2	11.7	17.8	6.5	10.0	14.7	8.1	13.5	20.6
Unsaturated Fat (g)	30-45^g^	22.2	34.7	51.3	19.3	29.8	42.9	27.0	41.9	62.7
Protein^b^ (g/kg)	0.71^ce^, 0.73^cf^	0.89	1.39	1.98	0.77	1.21	1.73	1.08	1.56	2.18
Vitamin A (µg RAE)	485^ce^, 630^cf^	221.1	429.9	737.7	183.7	387.4	648.0	275.4	499.5	834.2
Thiamine, B1 (mg)	0.9^ce^, 1.0^cf^	0.9	1.3	2.0	0.7	1.1	1.6	1.0	1.6	2.3
Riboflavin, B2 (mg)	0.9^ce^, 1.1^cf^	1.1	1.6	2.4	0.9	1.4	2.0	1.3	2.0	2.9
Niacin, B3 (mg)	11^ce^, 12^cf^	11.1	17.7	26.9	9.6	15.2	22.3	14.0	21.2	31.2
Vitamin B12 (µg)	2.0^c^	1.8	3.5	5.6	1.4	2.7	4.5	2.6	4.3	6.8
Vitamin C (mg)	56^ce^, 63^cf^	15.4	54.3	134.7	14.9	50.8	125.1	17.3	60.3	148.7
Vitamin D (µg)	10^c^	1.0	3.1	5.9	0.7	2.4	4.9	1.7	4.4	7.8
Folate (µg DFE)	330^c^	206.0	335.5	503.7	189.8	300.3	444.5	233.3	382.0	564.4
Calcium (mg)	1100^c^	421.7	736.5	1143.0	349.0	640.2	969.1	541.9	849.7	1362.8
Iron (mg)	7.9^ce^, 7.7^cf^	8.2	12.0	17.0	7.2	10.5	14.4	9.7	14.3	19.6
Zinc (mg)	7.3^ce^, 8.5^cf^	5.4	8.2	12.2	4.5	7.0	10.0	6.8	10.3	14.1
Sodium (mg)	2300^ h^	1448.5	2507.6	3775.4	1294.5	2197.9	3217.5	1857.4	3039.8	4409.9
Saturated fat (% total E)	< 10%^i^	8.7	11.6	14.2	8.3	11.3	13.9	9.3	12.2	14.4

*RAE* Retinol activity equivalent, *DFE* Dietary folate equivalent, *E* Energy

^a^Sugar includes naturally occurring and added sugars

^b^Protein requirements based on individual body size; total (*n* = 1289), females (*n* = 634), males (*n* = 602)

^c^Nutrients with an Estimated Average Requirement

^d^Nutrients with an Adequate Intake

^e^Recommendations for females

^f^Recommendations for males

^g^Canada Food Guide for Healthy Eating (2007)

^h^Nutrient with an Upper Limit

^i^World Health Organization guidelines for saturated fats

**Fig. 2:**
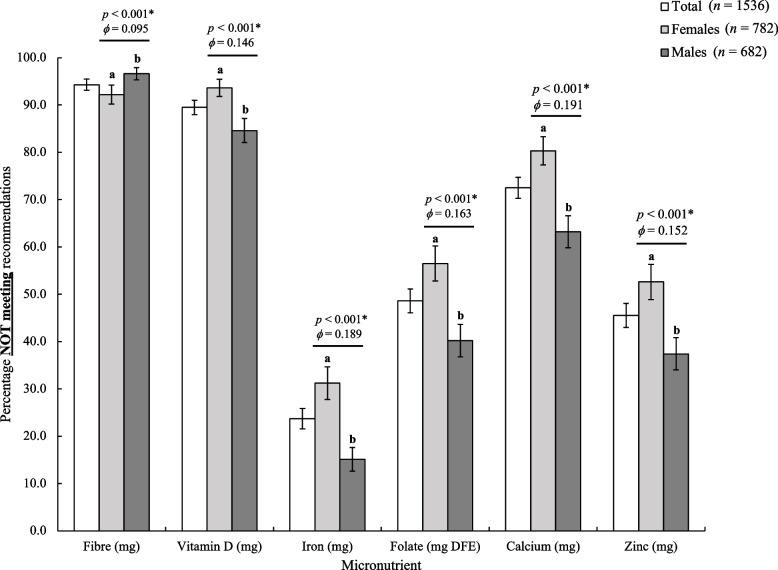
Percentage of participants not meeting recommendations for select nutrients. EAR used for all micronutrients except for fiber (AI). For Total, participants not reporting sex (*n* = 72) were excluded for comparison with guidelines that vary by sex (fibre, iron, zinc). Bars represent the percentage of participants not meeting recommendations; whiskers represent 95% confidence intervals. *p*-values and Cramer’s V are for comparisons between sexes. Bars not sharing a similar letter (a,b) denote significant differences (*p* < 0.05)

All study participants had adequate carbohydrate and protein intakes (Table 2). However, median fibre intake was inadequate for both sexes, with median intakes under half the recommended amount for males and just over half for females. Over 90% of respondents consumed inadequate fibre amounts (Fig. 2). Similarly, the majority of study participants consumed inadequate levels of vitamin D (90%) and calcium (70%) with a smaller proportion of males consuming inadequate levels of these nutrients compared to females. The majority of study participants consumed adequate levels of iron (75%). More than half of females did not meet recommendations for folate or zinc. Median sodium intakes exceeded recommendations for males but not for females, while median saturated fat intake as a percentage of total Energy exceeded recommendations for males and females. Average macronutrient distribution as proportion of total kilocalories for all participants was as follows: carbohydrates 52%; protein 16%; fats 32%.

### Food group intake

The majority of respondents did not meet Health Canada’s recommendations for food group servings (Table 3). A statistically significant higher proportion of females compared to males were not consuming recommended servings of Grain Products (46.5% vs. 38.4%) and Milk and Alternatives (80.3% vs. 66.9%). Study participants averaged 4.5 servings (males) and 3.4 servings (females) of Other Foods (including beverages) outside the EWCFG four food groups.

**Table 3:** Eating Well with Canada’s Food Guide (2007) food group servings, and percentage not meeting recommendations

Food group	*EWCFG* recommended servings	Total (*n* = 1536 recalls)		Females (*n* = 782 recalls)		Males (*n* = 682 recalls)		*p-*value	Cramer’s V
		Mean (SD)	% Not meeting recommendations	Mean (SD)	% Not meeting recommendations	Mean (SD)	% Not meeting recommendations		
Grain products	6^a^, 7^b^	7.3 (4.1)	42.8	6.4 (3.6)	46.5^c^	8.4 (4.4)	38.4^d^	0.002*	0.082
Vegetables and Fruit	7^a^, 8^b^	3.1 (2.6)	93.9	3.0 (2.4)	94.1^c^	3.2 (2.8)	93.5^c^	0.651	
Milk and Alternatives	3–4	2.1 (1.9)	74.0	1.7 (1.6)	80.3^c^	2.5 (2.2)	66.9^d^	< 0.001*	0.153
Meat and Alternatives	2^a^, 3^b^	2.4 (2.0)	57.0	2.0 (1.7)	56.6^c^	2.9 (2.1)	57.5^c^	0.749	
Other Foods	NA	3.9 (3.5)	NA	3.4 (3.1)	NA	4.5 (3.9)	NA		

*EWCFG* Eating Well with Canada’s Food Guide (2007), *NA* Not applicable

^a^Recommendations for females

^b^Recommendations for males

^c,d^Percentage values in a row with unlike letters are significantly different

^*^ *p* < 0.05: Pearson chi-square test for percentage not meeting recommendations by sex

**Fig. 3:**
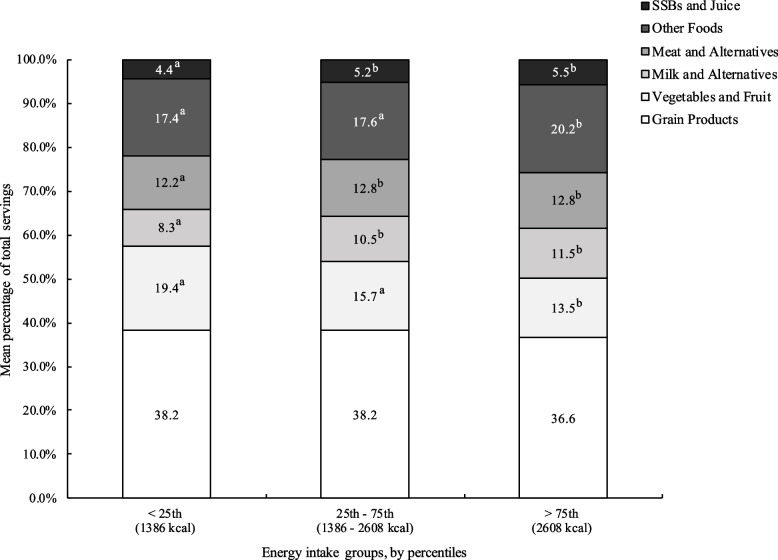
Mean percentage of food group servings contributing to total servings by energy intake group. Participant diet recalls were grouped according to whether their total caloric intake was below the 25^th^ (1386 kcal), between 25 and 75^th^ (1386–2608 kcal) or above 75^th^ (2608 kcal) percentiles. Bars not sharing a similar letter (a,b) for specific food group denote significant differences (*p* < 0.05). SSBs: sugar sweetened beverages

Figure 3 and Table 4 show that respondents who consumed the most calories (above the 75^th^ percentile in caloric intake distribution) obtained a significantly lower percentage of their total food group servings from Vegetables and Fruit (13.5%) and a higher percentage of servings from Other Foods (20.2%) and Sugar Sweetened Beverages and Juice (SSBs, 5.5%) than did respondents consuming the least calories (less than 25^th^ percentile). The same trend was observed for both males and females (Table 4).

**Table 4:** Mean percentage of food group servings contributing to total servings, by sex and energy intake group

Energy intake groups, by percentiles	Total				Females				Males			
	Calorie range	*n*	Mean (SD)	*p-*value	Calorie range	*n*	Mean (SD)	*p-*value	Calorie range	*n*	Mean (SD)	*p-*value
Grain products												
< 25	< 1386 kcal	384	38.2 (21.0)	0.169	< 1199 kcal	195	37.3 (21.5)	0.891	< 1736 kcal	170	39.2 (20.4)	0.153
25–75	1386–2608 kcal	767	38.2 (14.7)		1199–2248 kcal	391	37.9 (15.2)		1736–2916 kcal	342	38.1 (14.4)	
> 75	> 2608 kcal	385	36.6 (12.7)		> 2248 kcal	196	37.4 (12.1)		> 2916 kcal	170	35.7 (11.9)	
Vegetables and Fruit												
< 25	< 1386 kcal	384	19.4^a^ (17.1)	0.001*	< 1199 kcal	195	21.0^a^ (18.6)	0.042*	< 1736 kcal	170	15.3 (14.3)	0.517
25–75	1386–2608 kcal	767	15.7^a^ (11.0)		1199–2248 kcal	391	17.3^a,b^ (11.4)		1736–2916 kcal	342	14.3 (10.7)	
> 75	> 2608 kcal	385	13.5^b^ (8.7)		> 2248 kcal	196	15.1^b^ (8.6)		> 2916 kcal	170	12.6 (8.4)	
Milk and Alternatives												
< 25	< 1386 kcal	384	8.3^a^ (9.1)	< 0.001*	< 1199 kcal	195	7.2^a^ (8.8)	< 0.001*	< 1736 kcal	170	9.8^a^ (9.4)	0.003*
25–75	1386–2608 kcal	767	10.5^b^ (7.6)		1199–2248 kcal	391	10.1^b^ (7.7)		1736–2916 kcal	342	11.0^b^ (7.9)	
> 75	> 2608 kcal	385	11.5^b^ (7.8)		> 2248 kcal	196	10.7^b^ (6.8)		> 2916 kcal	170	12.0^b^ (7.4)	
Meat and Alternatives												
< 25	< 1386 kcal	384	12.2^a^ (13.0)	0.003*	< 1199 kcal	195	11.9^a^ (14.1)	0.019*	< 1736 kcal	170	13.7 (12.2)	0.372
25–75	1386–2608 kcal	767	12.8^b^ (9.7)		1199–2248 kcal	391	12.2^b^ (9.3)		1736–2916 kcal	342	13.2 (10.1)	
> 75	> 2608 kcal	385	12.8^b^ (8.7)		> 2248 kcal	196	12.3^b^ (9.1)		> 2916 kcal	170	13.5 (7.7)	
Other Foods												
< 25	< 1386 kcal	384	17.4^a^ (15.2)	< 0.001*	< 1199 kcal	195	17.9 (15.4)	0.159	< 1736 kcal	170	17.1^a^ (15.0)	0.009*
25–75	1386–2608 kcal	767	17.6^a^ (11.6)		1199–2248 kcal	391	17.8 (11.8)		1736–2916 kcal	342	18.0^a^ (11.4)	
> 75	> 2608 kcal	385	20.2^b^ (11.4)		> 2248 kcal	196	19.3 (11.4)		> 2916 kcal	170	20.4^b^ (11.3)	
Sugar-sweetened beverages and Juice												
< 25	< 1386 kcal	384	4.4^a^ (7.6)	< 0.001*	< 1199 kcal	195	4.7^a^ (8.1)	0.004*	< 1736 kcal	170	4.9^a^ (7.6)	0.006*
25–75	1386–2608 kcal	767	5.2^b^ (6.3)		1199–2248 kcal	391	4.6^a,b^ (5.8)		1736–2916 kcal	342	5.5^b^ (6.2)	
> 75	> 2608 kcal	385	5.5^b^ (5.9)		> 2248 kcal	196	5.2^b^ (5.8)		> 2916 kcal	170	5.9^b^ (6.1)	

^a,b^Mean percentage values in a column group with unlike letters are significantly different

^*^p < 0.05: Kruskal Wallis H test for mean percentages of food group servings

### Body mass index

Overall, 72.1% of students were classified as having a healthy BMI, with 25% classified as overweight or obese (Table 5). Significantly more males (9%) were classified as obese than females (5.9%). Four percent of males and 1.7% of females were classified as underweight.

**Table 5:** Body mass index classification

BMI Category (scoring criteria)	Total (*n* = 1242)^a^		Females (*n* = 630)		Males (*n* = 612)		*p-*value	Cramer’s V
	n	%	n	%	n	%		
Underweight (z-score < -2.0)	37	3.0	11^b^	1.7	26^c^	4.0	< 0.001*	0.121
Healthy weight (-2.0 ≤ z-score ≤ 1.0)	895	72.1	485^b^	77.0	410^c^	67.0		
Overweight (1.0 < z-score ≤ 2.0)	218	17.6	97^b^	15.4	12^c^	19.8		
Obese (z-score > 2.0)	92	7.4	37^b^	5.9	55^c^	9.0		

*BMI* Body Mass Index

^a^*n* = 1242 due to participants not reporting their height, weight, or sex

^b,c^Percentage values in a row with unlike letters are significantly different

^*^p < 0.05: Pearson chi-square test of association by sex

## Discussion

This study is the most comprehensive review of dietary intake among Manitoba youth undertaken to date with significant implications for upstream adolescent nutrition programs and policies.

Results clearly demonstrate that Manitoba youth are consuming low intakes of key nutrients and food groups required for healthy growthy and development, with concomitant high intakes of nutrients and foods of public health concern. These findings are consistent with dietary patterns of youth observed in other jurisdictions [23], and indicate that Manitoba youth have dietary patterns that are putting them at serious risk for the development of chronic nutrition-related diseases in adulthood. The observation that over 25% of study participants are already overweight or obese reinforces that the negative trajectory toward future chronic illness is already in place in Manitoba youth. There is a sense of urgency here, as NCDs such as type 2 diabetes are no longer relegated to older adulthood, and are increasing dramatically in adolescents and young adults [50]. In Winnipeg, Manitoba’s largest city, it is projected that 5330 0–19 year olds will have diagnosed or undiagnosed diabetes by 2032, an expected increase of 29% since 2015 [51]. The data reported in this study also indicate that youth may be at risk for developing poor mental health related to body image, as body weight is a significant factor in youth mental well-being due to societal pressures for young women and men to have flawless bodies and be hyper-fit [52–55].

### Food group intake

More than half of participants did not meet recommendations for food group intake the previous day for three out of four EWCFG food groups. Of particular concern, almost none of the participants consumed the recommended servings of Vegetables and Fruit, and three-quarters of females had inadequate Milk and Alternatives. Although mean intake of Vegetables and Fruit were below those reported from the 2015 Canadian Community Health Survey (CCHS; Nutrition, 13–17 years), servings for Grain Products, Milk and Alternatives, Meat and Alternatives were similar [56]. At the same time, participants consumed Other Foods and beverages outside the EWCFG four food groups [57], a similar pattern observed with adolescents elsewhere in Canada [24, 58, 59].

Students with higher energy intakes are obtaining additional kilocalories from less nutritious sources. This is not surprising, as foods such as salty snacks, candy and sweets, and sugar sweetened beverages are convenient, mostly ready-to-eat, and are among the foods most aggressively marketed to youth [60–62], due to their high differential profit margins [63]. In Canada, youth consume a significant number of calories from sugar sweetened beverages/juice [64].

### Energy and nutrient intakes

Energy intakes observed for both males and females were similar to those reported in the 2015 CCHS (Nutrition) [65]. Both male and female respondents in the lowest quartile reported very low energy intakes. This could be a result of measurement error, a well-established observation in self-reported dietary studies, which tend to underestimate energy intake, often due to reporting bias. The same trend was been observed in the CCHS (Nutrition), where the 2015 data showed an almost 200 kcal decrease over the previous 2004 survey [66]. The low-calorie intakes observed may also be indicative of widespread diet culture, including body image dissatisfaction and attempts at weight loss/restriction [67, 68]. More research is required to understand the relationships between diet culture and diet quality.

Intake of a number of key nutrients were observed to be inadequate for boys and/or girls including fibre, vitamin D, calcium, zinc and folate. Adequate intake of fibre and micronutrients are essential for optimal growth and development, as well as for reduction of risk for nutrition-related chronic diseases such as type 2 diabetes, heart disease and cancer [69–71]. Of particular concern for young women is low folate intake, as this is associated with increased risk of low birthweight and birth defects [72]. Low vitamin D and calcium consumption in Manitoba are of particular concern, as the high latitude and cold climate preclude cholecalciferol skin synthesis for much of the year, and vitamin D-fortified milk is prohibitively expensive in many northern and remote communities. At the same time, sodium and saturated fat intakes were above recommended upper limits for the majority of participants, noted in other Canadian dietary intake data [73, 74]. Notably, significantly more males did not meet recommendations for vitamin D, iron, folate, calcium and zinc. This suggests that, although their caloric needs and intake are higher, the nutritional quality of the foods eaten by the group is sub-optimal.

The dietary patterns shown in this study may be associated with the high rates of overweight and obesity observed in this study, which are consistent with the most recent national statistics reporting 6.2% of 12–17 year-olds having obesity and 16.9% having overweight [75]. Further research, however, is warranted to identify specific dietary patterns and their association to body weight.

The sub-optimal consumption of Vegetables and Fruits, and Milk and Alternatives in particular, as well as other healthy foods observed in this study, may be indicative of food insecurity in segments of the adolescent population. Food security is inversely associated with income [76]. Manitoba has the highest child poverty rate in Canada, at 31.6% (12% above the national average) [30]. This may be higher now due to the negative economic impacts of COVID-19 [77].

### Limitations and future research

There are several limitations to this research. First, the 24-h dietary recall and food frequency questions are self-reported and subject to recall error; inaccurate estimation of portion size; do not reflect day-to-day variability of individuals’ food intake; and may produce socially-desirable responses (e.g., reporting of less “junk” food and more “healthy” food) [78–81]. The web-based tool used for data collection, however, may have minimized the impact of some of these limitations through the use of graphic food images and prompts to enhance respondent memory [58]. Further, the 24-h recall method is an accepted and valid method for dietary assessment [82]. Second, some volunteer bias may be present, as not all schools participated, and parents needed to consent to having their child participate. Nonetheless, food patterns and nutrient intakes were similar to those observed in other Canadian regions, in similar populations. Finally, it is well-established that self-report measures yield bias, with height overestimated and weight underestimated [83]. Consequently, BMI results presented here may reflect underestimates of overweight and obesity. Despite these limitations, the FANS study presents an in-depth examination of adolescent nutrition, food behaviour and food security in a Canadian province, using a large sample. Further research is needed to examine food and eating behaviours, diet quality, and the role of food insecurity in dietary patterns, regional and other socio-demographic variations in dietary patterns. These will be examined in further research studies emerging from the FANS study. Additional studies will model linked administrative data to survey and dietary data.

## Conclusions

The dietary patterns observed in this study signal cause for concern: too many adolescents are consuming a diet that puts them at risk for future health issues. Indeed, the emergence of NCDs, such as type 2 diabetes in younger age cohorts suggests a crisis is upon us. Further, the COVID pandemic has revealed the insidious linkages between chronic and infectious disease, where obesity and metabolic risk factors are contributing to significant predisposing conditions now understood to increase the risk of serious illness and death due to COVID-19 and variants [84].

Major policy and program strategies must be investigated, championed at the highest levels, and funded to reverse the alarming trend in dietary patterns and other risk factors for nutrition-related illness. This could be undertaken by a provincial task force with stakeholders representing diverse sectors including education, health, social welfare and business. For too long the mantra of individual/parental responsibility, along with concerns about financial implications of population preventive strategies, have been the fall-back discourse of politicians and other key decision-makers. Considering that in 2016 alone, diagnosed, undiagnosed and pre-diabetes in the 20–34 year old Winnipeg population was estimated to cost over $14 million, a re-examination of strategic prevention approaches is clearly warranted [85].

## Data Availability

The datasets generated during and/or analysed during the current study are not publicly available due to the nature of the research and study participants not agreeing for their data to be shared with the public. Queries about data and materials should be directed to joyce.slater@umanitoba.ca.

## Abbreviations

NCDs: Noncommunicable diseases
FANS: Food and Nutrition Security for Manitoba Youth
WEB-Q: Waterloo Eating Behaviour Questionnaire
BMI: Body Mass Index
DRI: Dietary Reference Intakes
EAR: Estimated Average Requirement
AI: Adequate Intake
UL: Tolerable Upper Intake Levels
AMDR: Acceptable Macronutrient Distribution Range
EWCFG: Eating Well with Canada’s Food Guide
SSBs: Sugar Sweetened Beverages and Juice
CCHS: Canadian Community Health Survey
